# Stochastic Latency Guarantee in Wireless Powered Virtualized Sensor Networks

**DOI:** 10.3390/s21010121

**Published:** 2020-12-27

**Authors:** Ruyan Wang, Ailing Zhong, Zhidu Li, Hong Zhang, Xingjie Li

**Affiliations:** 1 School of Communication and Information Engineering, Chongqing University of Posts and Telecommunications, Chongqing 400065, China; wangry@cqupt.edu.cn (R.W.); lizd@cqupt.edu.cn (Z.L.); hongzhang@cqupt.edu.cn (H.Z.); 2018210145@stu.cqupt.edu.cn (X.L.); 2 Key Laboratory of Optical Communication and Networks, Chongqing 400065, China; 3 Key Laboratory of Ubiquitous Sensing and Networking, Chongqing 400065, China

**Keywords:** wireless virtualized sensor networks, wireless powered communications, effective capacity, latency guarantee, task allocation

## Abstract

How to guarantee the data rate and latency requirement for an application with limited energy is an open issue in wireless virtualized sensor networks. In this paper, we integrate the wireless energy transfer technology into the wireless virtualized sensor network and focus on the stochastic performance guarantee. Firstly, a joint task and resource allocation optimization problem are formulated. In order to characterize the stochastic latency of data transmission, effective capacity theory is resorted to study the relationship between network latency violation probability and the transmission capability of each node. The performance under the FDMA mode and that under the TDMA mode are first proved to be identical. We then propose a bisection search approach to ascertain the optimal task allocation with the objective to minimize the application latency violation probability. Furthermore, a one-dimensional searching scheme is proposed to find out the optimal energy harvesting time in each time block. The effectiveness of the proposed scheme is finally validated by extensive numerical simulations. Particularly, the proposed scheme is able to lower the latency violation probability by 11.6 times and 4600 times while comparing with the proportional task allocation scheme and the equal task allocation scheme, respectively.

## 1. Introduction

The rapid evolution of communication and intelligent technologies is inviting all human beings to the era of the Internet of everything, where unprecedented changes will have a profound impact on every single aspect of our daily interactions [1,2,3,4]. As a consequence, an exponentially increasing amount of data is needed to be sensed from different areas, which brings a large burden to the wireless sensor networks (WSNs). In this sense, virtualized WSN is proposed to manage the WSNs from different operators centrally with the objective of resource utilization improvement [5]. However, similar to the traditional WSNs, energy is one of the key factors bring performance bottlenecks to the virtualized WSNs. In addition to tag identification [6], radio frequency (RF) energy has been considered to be a stable energy source for wireless sensors. Moreover, wireless powered communication has attracted attention from both academia and industria [7,8]. Hence, it is a promising idea to integrate the wireless energy transfer (WET) technology into the virtualized WSNs, which is called wireless powered virtualized sensor networks.

Additionally, various types of Internet of Things applications are latency sensitive [9,10], where sensors are required to send data under different application latency requirements. Due to the time-varying wireless channel and large amount of sensing data, how to guarantee the latency requirement for different applications is worth studying in a WSN. Particularly, in a wireless powered virtualized sensor network, the data sensing task of an application is allocated to different sensor nodes with heterogeneous capabilities. Hence, latency guarantee in such type of network is more complex. To the best of our knowledge, a stochastic latency guarantee of wireless powered virtualized sensor networks is still an open problem.

Motivated by this, this paper studies a joint task and resource allocation scheme in a wireless powered virtualized sensor network under stochastic latency constraints. Firstly, a framework is constructed to integrate virtualized WSN and WET together, based on which an optimization problem is formulated with the objec tive of network latency violation probability (LVP) minimization. Then, effective capacity theory is applied to prove that identical latency performance can be guaranteed by the FDMA and TDMA modes in the considered network. Thereafter, a bisection search algorithm is proposed to determine the optimal task allocation scheme when system time configuration is given. Furthermore, the optimal energy harvesting time is obtained by a one-dimensional search scheme. Finally, insightful results are presented by numerical simulations. The main contributions of this paper are as follows:

- A three-layer architecture for wireless powered virtualized sensor network is proposed. Based on the proposed architecture, we prove that the FDMA mode can guarantee identical latency performance to the TDMA mode, when each node is allocated equal frequency resource or time resource.
- A joint task and resource allocation scheme is proposed to minimize the network latency violation probability. It is highlighted that the complexity of the proposed scheme is on a logarithmic level, which is applicable to the realistic engineering application.
- Numerical analysis reveals that the data rate requirement of an application and the number of sensor nodes both have linear or approximately linear impacts on the optimal energy harvesting time. This can be useful to quickly find out the optimal energy harvesting configuration in a practical network.

The remainder of this paper is organized as follows: Section 2 introduces the related works. Section 3 proposes a wireless powered virtualized sensor network model and communication model. The problem of the stochastic latency guarantee strategy based on effective capacity theory is formulated in Section 4, and the optimal solution is obtained in Section 5. In Section 6, we analyze the simulation results. Section 7 gives a discussion of our work and finally concludes the paper in Section 8.

## 2. Related Work

In order to operate multiple applications effectively, virtualization idea is introduced to WSNs at node level or network level [5,11]. Virtualization technology can improve the physical resource utilization of a WSN due to resource multiplexing among different applications. However, the contention of multiple applications for network resources also brings extra latency overhead to the WSNs. In the literature, related works about virtualized WSNs usually focus on network metric optimization, such as traffic throughput, energy efficiency, etc. In [12], an SDSense architecture was proposed to decompose the network functions into slow and fast changing components. Under the SDSense architecture, all the parameters of the sensors nodes could be reconfigured, such that the throughput of the considered WSN was improved. To reduce the data backlogs in a single-hop WSN, a uniforming random ordered policy (UROP) was proposed by Gul et al., where nearly optimal traffic throughput was obtained over a finite time horizon [13]. In addition, evolutionary game theory was applied to allocate data sensing load among different sensor nodes under the data rate requirement constraint of a certain application [14]. In [15], the application sensing task assignment problem was studied to maximize the overall energy consumption, where sensor nodes’ available energy and virtualization overhead were taken into account. In [16], the authors focused on energy efficiency maximization and then proposed a novel cyber-physical-social smart system. The authors therein employed wireless network virtualization to enhance the diversity and the flexibility of the service operation and the system management, and proposed a robust energy-efficient resource allocation scheme to outage probability requirements of controllers and actuators. Works [12,13,14,15,16] have provided insightful results on performance optimization in virtualized WSN. However, latency analysis is absent in those works. In order to find out the optimal trade-off between quality of service (QoS) (e.g., reliability) and Quality of Information (e.g., sensing accuracy), an offline embedding algorithm that searches through all possible embedding was proposed in [17]. In this regard, the search time can be controlled intuitively according to the application requirements.

Recently, RF energy harvesting is considered as a promising technology for wireless power sensors that are energy limited [18]. In the literature, wireless powered sensor networks have attracted attention from the academia. In [19], simultaneous wireless information and the power transfer (SWIPT) technique were introduced to a mobile WSN where energy harvest by relay nodes can compensate their energy consumption on data forwarding. A cross-layer resource allocation scheme was proposed to maximize the energy efficiency under different scenarios. Aiming at improving energy efficiency for a TDMA based wireless energy harvesting sensor network, Ref. [20] proposes a scheme to optimize the system time allocation and transmission power configuration. In [21], an adaptive multi-sensing (MS) framework was proposed, where each node was mounted with heterogeneous sensors to sense multiple cross-correlated slowly-varying parameters/signals. To increase the energy efficiency, a network and node-level collaborations based multi-sensing scheme was studied to deal with a formulated multi-objective optimization problem that jointly takes sensing quality and network energy efficiency into account. Ref. [22] focused on system sum throughput maximization of the considered sensor network, where two scenarios were considered, i.e., multiantenna power station and the sensor nodes belong to the same or different service operator(s). The authors therein proposed two different schemes to optimize the system time and energy harvesting rate for the two scenarios, respectively. Similar to works [12,13,14,15,16], works [19,20,21,22] also aimed to optimize the energy efficiency or network throughput for a WSN. How to guarantee the application latency was still unknown.

In other wireless networks, such as Internet of Vehicles and mobile cellular networks, latency or delay analysis can be resorted to the effective capacity theory [23]. With consideration of the time-varying channel gain, the maximum traffic rate that can be sustained by a vehicle-to-vehicle (V2V) link was studied in [24], based on which, the latency violation probability of the V2V link can be deduced. Additionally, the aggregate effective capacity was derived for heterogeneous statistical QoS provisioning in a wireless powered sensor network [25]. Particularly, the aggregate effective capacity was maximized by solving the hybrid access point determined downlink energy assignment problem and the sensor node determined uplink power control problem, where the optimal system time allocation, the downlink energy assignment, and uplink power transmission were obtained. Meanwhile, network calculus is considered as a powerful tool in end-to-end performance analysis of wireless communication networks [26]. In [27], a network calculus based framework was constructed to guarantee the delay bound and the target reliability of each application for industrial WSNs with consideration of low-power communications and the harsh wireless environment. However, task allocation was not considered in [25,26,27].

In summary, how to allocate application tasks to the sensors under the latency requirement is still an open problem, which motivates this paper.

## 3. System Model

### 3.1. Network Model

**Figure 1:**
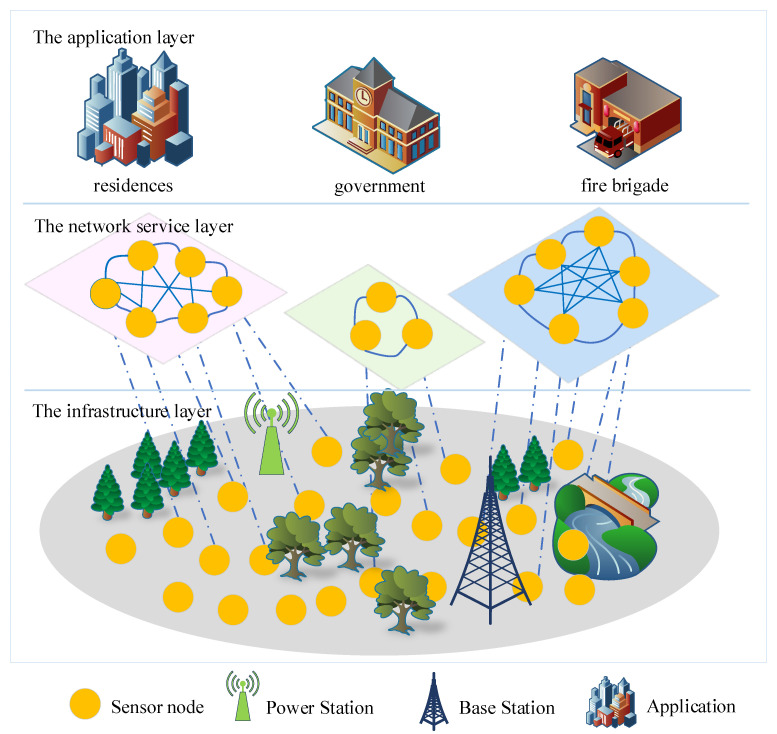
Network model of wireless powered virtualized sensor networks.

In a wireless powered virtualized sensor network, the network service provider can rent node resources to different applications through the network virtualization technique. As depicted in Figure 1, the considered wireless powered virtualized sensor network consists of three layers, i.e., the infrastructure layer, the network service layer, and the application layer. More specifically, the infrastructure layer consists of a power station (PS), a base station (BS), and sensor nodes (SNs). The network service layer contains multiple VSNs constructed by operator, and the nodes in a VSN can communicate with each other. The application layer contains multiple applications that need data sensed from the infrastructure layer under given latency requirements. When an application initiates a request, the operator selects appropriate SNs, donated by $SN=\{SN_{1},SN_{2},\cdots ,SN_{K}\}$ to form a VSN. The corresponding tasks are assigned to the physical nodes mapped by the virtual nodes. Then, the application data request is completed by the *K* nodes cooperatively under the given latency requirement.

### 3.2. Communication Model

**Figure 2:**
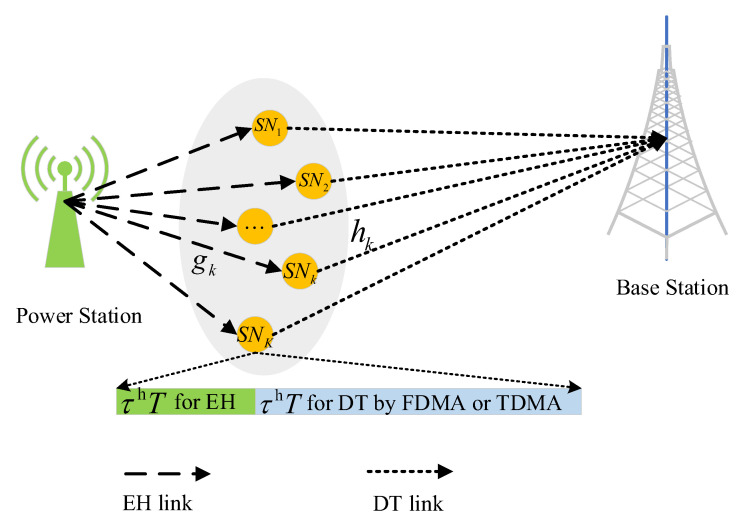
Communication model of the sensor node.

The detailed infrastructure layer model is depicted in Figure 2. The total network bandwidth is denoted by $B_{\mathrm{tot}}$. The system time is divided into several time blocks with equal duration *T*. Each time block contains both the downlink energy harvesting (EH) process and the uplink data transmission (DT) process. In the EH process, the PS transmits RF signals to all SNs with a duration of $\tau^{h}T$. In the DT process with duration $\tau^{t}T$, each SN uses the harvested energy to transmit the sensing data to the BS cooperatively through FDMA or TDMA modes. Here, the DT duration and bandwidth for $SN_{k}$ are denoted by $\tau_{k}^{t}$ and $B_{k}$, respectively. Specifically, in FDMA mode, the bandwidth are equally allocated to each node while the DT time of each node is equal to $\tau^{t}T$, i.e.,

(1) $$B_{k}=\frac{B_{\mathrm{tot}}}{K},\;\tau_{k}^{t}=\tau^{t}.$$

In TDMA mode, each node can share the whole bandwidth while the DT time is equally allocated to each node, i.e.,

(2) $$B_{k}=B_{\mathrm{tot}},\;\tau_{k}^{t}=\frac{\tau^{t}}{K}.$$

Both uplink and downlink channels are assumed to be quasi-static flat fading. We use $g_{k}$ to represent the channel gain due to small-scale fading between PS and $SN_{k}$, and $h_{k}$ to represent the one between $SN_{k}$ and BS. The values of $g_{k}$ and $h_{k}$ remain unchanged during a time block while the values in different time blocks follow identically and independently distribution (i.i.d).

## 4. General Optimization Framework

Denote the transmission power of the PS by $p_{0}$; ignoring the influence of background noise on energy collection, the received RF energy of $SN_{k}$ in the *i*-th time block holds as:

(3) $$P_{k}^{\mathrm{RF}}\left(i\right)=p_{0}g_{k}\left(i\right)l_{k}^{h},$$

where $l_{k}^{h}$ is the path loss between PS and $SN_{k}$, which depends on the distance between the PS and $SN_{k}$.

The harvested RF energy needs to be converted into DC energy before it can be using by SNs. In order to better characterize the realistic RF energy conversion circuit, this paper adopts a nonlinear energy conversion model. In this model, the rate of DC energy collected by $SN_{k}$ in the *i*-th time block can be obtained as:

(4) $$P_{k}^{\mathrm{DC}}\left(i\right)=\pi_{k}\frac{1-e^{-v_{k}P_{k}^{\mathrm{RF}}\left(i\right)}}{1+e^{-v_{k}\left(P_{k}^{\mathrm{RF}}\left(i\right)-\gamma_{k}\right)}},$$

where parameters $\pi_{k}$, $v_{k}$, and $\gamma_{k}$ describe the nonlinear characteristics in the process of converting RF energy into DC energy due to the limitation of circuit hardware. Specifically, $\pi_{k}$ represents the maximum energy conversion rate, and $v_{k}$ and $\gamma_{k}$ denote the circuit sensitivity and current leakage, respectively. The specific values can be obtained by fitting the relevant data of the actual energy conversion circuit [28,29]. The energy harvested by $SN_{k}$ holds as:

(5) $$P_{k}\left(i\right)=P_{k}^{\mathrm{DC}}\left(i\right)\tau^{h}T,$$

The harvested energy is assumed to used up for uplink DT, i.e., the transmission power holds as:

(6) $$p_{k}\left(i\right)=\frac{P_{k}\left(i\right)}{\tau_{k}^{t}T}=\frac{P_{k}^{\mathrm{DC}}\left(i\right)\tau^{h}}{\tau_{k}^{t}}.$$

According to Shannon’s theorem, the date transmission rate in the *i*-th time block holds as:

(7) $$R_{k}\left(i\right)=B_{k}\log_{2}\left(1+\frac{p_{k}\left(i\right)h_{k}\left(i\right)l_{k}^{t}}{N_{0}B_{k}}\right),$$

where $l_{k}^{t}$ represents the path loss between $SN_{k}$ and BS, $N_{0}$ denotes the power spectral density of white Gaussian noise. Because the service process $\left\{R_{k}\left(i\right),i=1,2\cdots \right\}$ is not related between time slots, the effective capacity of $SN_{k}$ can be expressed as [30]:

(8) $$C_{k}\left(\theta_{k}\right)=-\frac{1}{\theta_{k}T}\ln E\left[e^{-\theta_{k}\tau_{k}^{t}TB_{k}\log_{2}\left(1+\frac{p_{k}\left(i\right)h_{k}\left(i\right)l_{k}^{t}}{N_{0}B_{k}}\right)}\right],$$

where E[·] denotes an expectation function, $\theta_{k}$ denotes the latency exponent of $SN_{k}$. In [30], it is proved that $C_{k}\left(\theta_{k}\right)$ is monotonically decreasing with $\theta_{k}$, i.e.,

(9) $$\left\{\begin{array}{c} C_{k}\left(\theta_{k}=0\right)=E[B_{k}\log_{2}\left(1+\frac{p_{k}\left(i\right)h_{k}\left(i\right)l_{k}^{t}}{N_{0}B_{k}}\right)],\\ C_{k}\left(\theta_{k}=\infty \right)=0. \end{array}\right.$$

In other words, when $\theta_{k}=0$, the network does not need to guarantee the LVP. Additionally, a tighter LVP requires larger $\theta_{k}$. Specifically, for a delay requirement $D_{\max}$ which is the maximum data latency tolerance for an application, the LVP of the *k*-th SN holds as:

(10) $$\mathrm{Pr}\left\{D_{k}>D_{\max}\right\}=\mathrm{Pr}\left\{Q_{k}>0\right\}e^{-\theta_{k}C_{k}\left(\theta_{k}\right)D_{\max}},$$

where $\mathrm{Pr}\{Q_{k}>0\}$ denotes the probability that the buffer $Q_{k}$ of the *k*-th SN is nonempty in the steady state. For a system, the busy period is more worthy of being focused on, thus we assume $\mathrm{Pr}\{Q_{k}>0\}=1$. In addition, according to the effective capacity theory, the maximum traffic rate of *k*-th SN that can be supported holds as $\lambda_{k}=C\left(\theta_{k}\right)$.

Let λ denote the data rate requirement of the application. It is interesting to investigate how to guarantee the minimum LVP for such application through optimizing the network parameters such as EH duration, DT duration, and task allocation. Furthermore, the network LVP, i.e., $\mathrm{Pr}\{D>D_{\max}\}$, is equal to the maximum LVP of the cooperative SNs. Hence, the optimization problem can be expressed as P1:

(11) $$\begin{array}{c} \begin{array}{ccc} \min & \max_{\lambda ,\tau^{h},\tau^{t}} & \mathrm{Pr}\left\{D_{k}>D_{\max}\right\},k\in \left\{1,2,\cdots ,K\right\}\\ s.t. & C1: & \lambda_{1}+\lambda_{2}+\cdots +\lambda_{K}\geq \lambda \\ & C2: & p_{k}\left(i\right)\leq p_{k}^{\max},\forall i\\ & C3: & \tau^{h}+\tau^{t}\leq 1\\ & C4: & \lambda_{k}=C_{k}\left(\theta_{k}\right) \end{array} \end{array}$$

where C1 ensures the source rate required by the application. Constraint C2 means the transmission power of a SN should be controlled within a maximum level. Constraint C3 means that the sum of EH duration and DT duration cannot exceed the duration of a time block. Constraint C4 reveals the relationship between the maximum sustained traffic rate and the effective capacity for a node.

## 5. Stochastic Latency Guarantee

In order to dealing with problem P1, we are interested in the difference of performance guarantee between the FDMA mode and TDMA mode. Surprisingly, if time and frequency resources are allocated equally to each SN, we can prove that the LVP performance of such two modes are identical, which is summarized in the following.

**Theorem** **1.** *The network LVP with FMDA mode is equal to the one with TDMA mode.*

**Proof.**  According to Equations (1), (6), and (8), we have the effective capacity for the FDMA mode as:

(12) $$\begin{array}{cc} C_{k}^{\mathrm{FDMA}}\left(\theta_{k}\right) & =-\frac{1}{\theta_{k}T}\ln E\left[e^{-\theta_{k}\tau_{k}^{t}TB_{k}\log_{2}\left(1+\frac{p_{k}h_{k}l_{k}^{t}}{N_{0}B_{k}}\right)}\right]\\ \; & =-\frac{1}{\theta_{k}T}\ln E\left[e^{-\theta_{k}\tau^{t}T\frac{B_{\mathrm{tot}}}{K}\log_{2}\left(1+K\frac{P_{k}^{\mathrm{DC}}\tau^{h}h_{k}l_{k}^{t}}{\tau^{t}N_{0}B_{\mathrm{tot}}}\right)}\right] \end{array}.$$

According to Equations (2), (6) and (8), we have the effective capacity for the TDMA mode as:

(13) $$\begin{array}{cc} C_{k}^{\mathrm{TDMA}}\left(\theta_{k}\right) & =-\frac{1}{\theta_{k}T}\ln E\left[e^{-\theta_{k}\tau_{k}^{t}TB_{k}\log_{2}\left(1+\frac{p_{k}h_{k}l_{k}^{t}}{N_{0}B_{k}}\right)}\right]\\ \; & =-\frac{1}{\theta_{k}T}\ln E\left[e^{-\theta_{k}\frac{\tau^{t}}{K}TB_{\mathrm{tot}}\log_{2}\left(1+K\frac{P_{k}^{\mathrm{DC}}\tau^{h}h_{k}l_{k}^{t}}{\tau^{t}N_{0}B_{\mathrm{tot}}}\right)}\right] \end{array}.$$

Comparing Equations (12) and (13), we have $C_{k}^{\mathrm{FDMA}}\left(\theta_{k}\right)=C_{k}^{\mathrm{TDMA}}\left(\theta_{k}\right)$. According to Equation (10), the LVP based on FDMA is equal to that based on TDMA for any SN when other parameters are fixed. As a result, the network LVPs based on such two modes are identical, which proves Theorem 1.  ☐

Based on Theorem 1, the solutions of problem P1 under the FDMA and TDMAs are identical. Additionally, the effective capacity of each SN is related to latency exponent $\theta_{k}$, which further affects the LVP performance according to Equation (10). The following theorem will reveal the relationship between the LVP performance and $\theta_{k}$.

**Theorem** **2.** *The LVP of a node decreases as the latency exponent $\theta_{k}$ increases.*

**Proof.**  According to Equations (8) and (10), we have

(14) $$\begin{array}{c} \mathrm{Pr}\{D_{k}>D_{\max}\}\\ =e^{-\theta_{k}\left(-\frac{1}{\theta_{k}T}\ln E\left[e^{-\theta_{k}\tau_{k}^{t}TB_{k}\log_{2}\left(1+\frac{p_{k}\left(i\right)h_{k}\left(i\right)l_{k}^{t}}{N_{0}B_{k}}\right)}\right]\right)D_{\max}}\\ =e^{\frac{1}{T}\ln E\left[e^{-\theta_{k}\tau_{k}^{t}TB_{k}\log_{2}\left(1+\frac{p_{k}\left(i\right)h_{k}\left(i\right)l_{k}^{t}}{N_{0}B_{k}}\right)}\right]D_{\max}} \end{array}.$$

It is easily verified that the LVP of $SN_{k}$ decreases as $\theta_{k}$ increases, which completes the proof of Theorem 2.  ☐

Based on Theorem 2, smaller $\theta_{k}$ can guarantee lower LVP performance for a SN. However, as mentioned before, smaller $\theta_{k}$ results in smaller effective capacity, which further decreases the sustained source rate for a SN. Hence, a trade-off between the LVP performance and the sustained source rate should be taken into account. In detail, for an arbitrary cooperative node $SN_{a}$ with data rate requirement $\lambda_{a}$, according to constraint C4 in Problem P1 and Equation (8), we can obtain the optimal $\theta_{a}$ by solving the following equation:

(15) $$f_{1}\left(\theta_{a}\right)=-\frac{1}{\theta_{a}T}\ln E\left[e^{-\theta_{a}\tau_{a}^{t}TB_{a}\log_{2}\left(1+\frac{p_{a}\left(i\right)h_{a}\left(i\right)l_{a}^{t}}{N_{0}B_{a}}\right)}\right]-\lambda_{a}.$$

As $\lambda_{a}$ is fixed and $C_{a}\left(\theta_{a}\right)$ decreases with $\theta_{a}$, $f_{1}\left(\theta_{a}\right)$ is a decreasing function $\theta_{a}$. Consequently, Equation (15) can be solved by the resorting bisection searching approach, which is summarized in the following. Note that, for a fixed calculation precision $\varepsilon_{\theta }$, the calculation complexity of Algorithm 1 holds as $O(\log_{2}\left(\frac{1}{\varepsilon_{\theta }}\right))$.
**Algorithm 1** Find optimal $\theta_{a}^{*}$1:**Input:**$\lambda_{a}$, $\theta_{a}^{\min}=0$ and $\theta_{a}^{\max}=1$, precision $\varepsilon_{\theta }$2:**Output:**$\theta_{a}^{*}$3:Compute $f_{1}\left(\theta_{a}^{\min}\right)$, $f_{1}\left(\theta_{a}^{\max}\right)$ by Equation (15).4:**while**$\left(f_{1}\left(\theta_{a}^{\min}\right)f_{1}\left(\theta_{a}^{\max}\right)<0\&\&\left(\theta_{a}^{\max}-\theta_{a}^{\min}\right)>\varepsilon_{\theta }\right)$**do**5: Set middle point θamid=(θamin+θamax)(θamin+θamax)22.6: Compute $f_{1}\left(\theta_{a}^{\mathrm{mid}}\right)$ by Equation (15).7: **if**
$\left(f_{1}\left(\theta_{a}^{\mathrm{mid}}\right)>0\right)$
**then**8:  $\theta_{a}^{\min}=\theta_{a}^{\mathrm{mid}}$.9: **else**10:  $\theta_{a}^{\max}=\theta_{a}^{\mathrm{mid}}$.11: **end if**12: Compute $f_{1}\left(\theta_{a}^{\min}\right)$, $f_{1}\left(\theta_{a}^{\max}\right)$ by Equation (15).13:**end while**14:$\theta_{a}^{*}=\left(\theta_{a}^{\min}+\theta_{a}^{\max}\right)/2$.15:**END**

According to Equation (11), problem P1 is a min-max problem. Hence, the relationship among the LVP of each SN should be addressed. The following theorem illustrates how to balance the LVP of each SN to obtain the optimal task allocation when system time allocation is given.

**Theorem** **3.** *When optimal task allocation is obtained as $\left\{\lambda_{1},...,\lambda_{K}\right\}$, then, for ∀m,n∈{1,2,⋯,K}(m≠n), there always holds:*

$$\mathrm{Pr}\left\{D_{n}>D_{\max}\right\}=\mathrm{Pr}\left\{D_{m}>D_{\max}\right\}.$$

**Proof.**  We prove Theorem 3 with a contradiction approach. Assume that, when optimal task allocation is obtained, there still exist the maximum LVP $\mathrm{Pr}\{D_{m}>D_{\max}\}$ for $SN_{m}$ and the minimum LVP $\mathrm{Pr}\{D_{n}>D_{\max}\}$ for $SN_{n}$, where m,n∈{1,2,⋯,K} and $\mathrm{Pr}\left\{D_{m}>D_{\max}\right\}>\mathrm{Pr}\left\{D_{n}>D_{\max}\right\}$, i.e., the assumed optimal task allocation solution is obtained under $\mathrm{Pr}\{D_{m}>D_{\max}\}$. In this case, the corresponding source rate for such two nodes are denoted by $\lambda_{m}$ and $\lambda_{n}$, respectively. In addition, the corresponding latency exponents for $SN_{m}$ and $SN_{n}$ at this time are denoted by $\theta_{m}$ and $\theta_{n}$, respectively. According to Theorem 2, there holds $\theta_{m}<\theta_{n}$. As the effective capacity decreases with the latency exponent, we have $\lambda_{m}>\lambda_{n}$.

Let ${\lambda_{m}}^{\prime }=\lambda_{m}-\Delta \lambda$, ${\lambda_{n}}^{\prime }=\lambda_{n}+\Delta \lambda$. We have $\theta_{m}<{\theta_{m}}^{\prime }$ and $\theta_{n}>{\theta_{n}}^{\prime }$. Furthermore, when $\Delta \lambda \to 0^{+}$, the constraint conditions in P1 are still satisfied. According to Theorem 2, we can obtain that

$$\begin{array}{cc} \mathrm{Pr}\{D_{m}>D_{\max}\} & >\mathrm{Pr}\{{D_{m}}^{\prime }>D_{\max}\}\\ \; & >\mathrm{Pr}\left\{{D_{n}}^{\prime }>D_{\max}\right\}>\mathrm{Pr}\left\{D_{n}>D_{\max}\right\} \end{array}.$$

Hence, the network LVP can be further reduced to $\mathrm{Pr}\{{D_{m}}^{\prime }>D_{\max}\}$, which brings the contradiction. Therefore, when optimal task allocation is obtained, the LVP of each SN should be equal to each other, which completes the proof.  ☐

In order to quickly ascertain the task allocation for each SN, the following corollary is given.

**Corollary** **1.** *When the source rate of a SN is allocated as $\lambda_{a}$, the source rate for the other nodes $SN_{k}$ can be obtained by solving the following equation:*

(16) ΔPr(a)=ΔPr(k),

*where*

(17) $$\Delta \mathrm{Pr}\left(k\right)=E\left[e^{-\theta_{k}\tau_{k}^{t}TB_{k}\log_{2}\left(1+\frac{p_{k}\left(i\right)h_{k}\left(i\right)l_{k}^{t}}{N_{0}B_{k}}\right)}\right].$$

*Note that ΔPr(k) is related to $\theta_{k}$; hence, we can construct a function as follows:*

(18) $$f_{2}\left(\theta_{k}\right)=E\left[e^{-\theta_{k}\tau_{k}^{t}TB_{k}\log_{2}\left(1+\frac{p_{k}\left(i\right)h_{k}\left(i\right)l_{k}^{t}}{N_{0}B_{k}}\right)}\right]-\Delta \mathrm{Pr}\left(a\right).$$

It is easily verified that $f_{2}\left(\theta_{k}\right)$ is a decreasing function of $\theta_{k}$. Hence, the solution $\theta_{k}^{*}$ of $f_{2}\left(\theta_{k}\right)=0$ can be obtained by a bisection search approach. Furthermore, the corresponding source rate $\lambda_{k}^{*}$ can be calculated by $\lambda_{k}^{*}=C\left(\theta_{k}^{*}\right)$. The method for task allocation is summarized in Algorithm 2. The computation complexity of Algorithm 2 holds as $O(\left(K-1\right)\log_{2}\left(\frac{1}{\varepsilon_{\theta }}\right))$.
**Algorithm 2** Task allocation scheme1:**Input:**ΔPr(a), precision $\varepsilon_{\theta }$, $\theta_{k}^{\min}=0$ and $\theta_{k}^{\max}=1$2:**Output:**$\lambda_{k}^{*},\left(k=\left\{1,2,3,\cdots ,K\right\}\&\&k!=a\right)$3:**for**(k={1,2,3,⋯,K}&&k!=a)**do**4: Compute $f_{2}\left(\theta_{k}^{\min}\right)$, $f_{2}\left(\theta_{k}^{\max}\right)$ by Equation (18).5: **while**
$\left(f_{2}\left(\theta_{k}^{\min}\right)f_{2}\left(\theta_{k}^{\max}\right)<0\&\&\left(\theta_{k}^{\max}-\theta_{k}^{\min}\right)>\varepsilon_{\theta }\right)$
**do**6:  Set middle point θkmid=(θkmin+θkmax)(θkmin+θkmax)22.7:  Compute $f_{2}\left(\theta_{k}^{\mathrm{mid}}\right)$ by Equation (18).8:  **if**
$\left(f_{2}\left(\theta_{k}^{\mathrm{mid}}\right)>0\right)$
**then**9:  $\theta_{k}^{\min}=\theta_{k}^{\mathrm{mid}}$.10:  **else**11:   $\theta_{k}^{\max}=\theta_{k}^{\mathrm{mid}}$.12:  **end if**13:  Compute $f_{2}\left(\theta_{k}^{\min}\right)$, $f_{2}\left(\theta_{k}^{\max}\right)$ by Equation (18).14: **end while**15: $\theta_{k}^{*}=\left(\theta_{k}^{\min}+\theta_{k}^{\max}\right)/2$.16: Compute $\lambda_{k}^{*}$ by C4 in Equation (11).17:**end for**18:**END**

According to Theorems 2 and 3, $\lambda_{k}$ and $\theta_{k}$ can be obtained when system time allocation is given. In the subsequence, a optimal system time allocation condition is given.

**Theorem** **4.** *To guarantee the minimum network LVP, the system time should be used up for energy harvesting and data transmission in each time block, i.e.,*

(19) $$\tau^{h}+\tau^{t}=1.$$

**Proof.**  Assume that $\left\{\tau^{h*},\tau^{t*}\right\}$ can guarantee the minimum LVP with $\tau^{h*}+\tau^{t*}<T$. Accordingly, we can construct another time allocation solution $\left\{{\tilde{\tau }}^{h},{\tilde{\tau }}^{t}\right\}$ which satisfying ${\tilde{\tau }}^{h}=\tau^{h*}+\Delta \tau^{h}>\tau^{h*}$ and ${\tilde{\tau }}^{t}=\tau^{t*}$, where $\Delta \tau^{h}=1-\left(\tau^{h*}+\tau^{t*}\right)$, i.e., ${\tilde{\tau }}^{h}+{\tilde{\tau }}^{t}=1$. In this case, the LVP is denoted by $\tilde{\mathrm{Pr}}$. It is easy to verify that $\left\{{\tilde{\tau }}^{h},{\tilde{\tau }}^{t}\right\}$ still satisfies all the constraints of problem P1, so it is a feasible solution. Additionally, when ${\tilde{\tau }}^{h}>\tau^{h*}$, each cooperative SN can harvest more energy, which implies that higher transmission power can be provided in the DT process. Hence, the effective capacity of SNs can be enhanced, which further reduces the network LVP. As a result, there is a contradiction and the system time should be used up for each time block.  ☐

Based on a similar idea of Theorem 4, we can also prove that, in order to guarantee the minimum LVP with $\tau^{h*}+\tau^{t*}<T$, there holds:

$$\lambda =\lambda_{1}+\lambda_{2}+\cdots +\lambda_{K}.$$

In all, problem P1 can be transferred to problem P2 as follows:

(20) $$\begin{array}{ccc} \; & \min_{\lambda ,\tau^{h},\tau^{t}} & \mathrm{Pr}\left\{D_{k}>D_{\max}\right\},k\in \left\{1,2,\cdots ,K\right\}\\ s.t. & C1: & \lambda_{1}+\lambda_{2}+\cdots +\lambda_{K}=\lambda \\ & C2: & p_{k}\left(i\right)\leq p_{k}^{\max},\forall i\\ & C3: & \tau^{h}+\tau^{t}=1\\ & C4: & \lambda_{k}=C_{k}\left(\theta_{k}\right)\\ & C5: & \mathrm{Pr}\left\{D_{k}>D_{\max}\right\}=\mathrm{Pr}\left\{D_{a}>D_{\max}\right\} \end{array}$$

In Algorithms 1 and 2, task allocation for one node, i.e., $SN_{a}$ is needed. Hence, we can fixed the system allocation and find out $\lambda_{a}$ firstly. Note that $\mathrm{Pr}\{D_{a}>D_{\max}\}$ is monotonically decreasing with $\lambda_{a}$ and $C_{a}\left(\theta_{a}\right)$ is monotonically decreasing with $\theta_{a}$, and there is a unique solution of λ for problem P2. Hence, the bisection search approach can be applied again. Furthermore, as the statistical channel information is different among all the SNs, according to Equation (8), an SN with poorer channel information guarantees lower effective capacity, which leads to a lower sustained source rate. In order to reduce the computation complexity of task allocation, we can choose the node with poorest statistical channel information as $SN_{a}$. In this case, the upper bound of the bisection search can be just $\frac{\lambda }{K}$. The following algorithm summarizes how to find out $\lambda_{a}$. It is easily verified that the computation complexity of Algorithm 3 lies in $O(K\log_{2}\left(\frac{\lambda }{\varepsilon_{\lambda }}\right)\log_{2}\left(\frac{1}{\varepsilon_{\theta }}\right))$.
**Algorithm 3** Find optimal $\lambda_{a}^{*}$1:**Input:**$\tau^{h}$, $p_{0}$, $N_{0}$, *W*, $g_{k}$, $h_{k}$, $\pi_{k}$, $v_{k}$, $\gamma_{k}$, $D_{\max}$, *K*, *T*, λ, $\lambda_{a}^{\min}=0$ and $\lambda_{a}^{\max}=\frac{\lambda }{K}$, precision $\varepsilon_{\lambda }$.2:**Output:**$\lambda_{a}^{*}$3:Compute $\lambda_{a}^{\mathrm{mid}}=\frac{\lambda_{a}^{\max}+\lambda_{a}^{\min}}{2}$.4:Apply Algorithm 1 to find out $\theta_{\min}$.5:Apply Algorithm 2 to find out $\lambda^{\mathrm{mid}}$.6:**while**$|\sum \lambda^{\mathrm{mid}}-\lambda |>\varepsilon_{\lambda }$**do**7: **if**
$\sum \lambda^{\mathrm{mid}}-\lambda >0$
**then**8:  $\lambda_{a}^{\max}=\lambda_{a}^{\mathrm{mid}}$.9: **else**10:  $\lambda_{a}^{\min}=\lambda_{a}^{\mathrm{mid}}$.11: **end if**12: $\lambda_{a}^{\mathrm{mid}}=\frac{\lambda_{a}^{\max}+\lambda_{a}^{\min}}{2}$.13: Apply Algorithm 1 to find out $\theta_{\min}$.14: Apply Algorithm 2 to find out $\lambda^{\mathrm{mid}}$.15:**end while**16:$\lambda_{a}^{*}=\left(\lambda_{a}^{\min}+\lambda_{a}^{\max}\right)/2$.17:**END**

According to Constraint 4 of problem P2, the optimal system time can be further obtained through one-dimensional search. Therefore, problem P2 can be solved. The procedure for solving the P2 is summarized in Algorithm 4. In all, the computation complexity of the proposed joint task and resource allocation scheme holds as $O(\frac{K}{\varepsilon_{\tau }}\log_{2}\left(\frac{\lambda }{\varepsilon_{\lambda }}\right)\log_{2}\left(\frac{1}{\varepsilon_{\theta }}\right))$.
**Algorithm 4** System time allocation scheme1:**Input:**$D_{\max}$, precision $\varepsilon_{\tau }$2:**Output:**$\tau^{h*}$, $\tau^{t*}$, $\min\mathrm{Pr}\{D>D_{\max}\}$3:**for** all $\tau^{h}$
**do**4:  **switch** TD mode **do**5:   **case:** TDMA6:      $\tau_{k}^{t}=\left(1-\tau^{h}\right)/K$.7:      $B_{k}=W$.8:   **case:** FDMA9:      $\tau_{k}^{t}=1-\tau^{h}$.10:      $B_{k}=W/K$.11:  **end switch**12:Apply Algorithm 3 to find out optimal $\lambda_{a}$ for $\tau^{h}$.13:Compute $\mathrm{Pr}\{D>D_{\max}\}$ according to Equation (10) and Constraint 4 of problem P2.14:**end for**15:Find out $\min\mathrm{Pr}\{D>D_{\max}\}$ and the corresponding $\tau^{h*}$, $\tau^{t*}$.16:**END**

**Figure 3:**
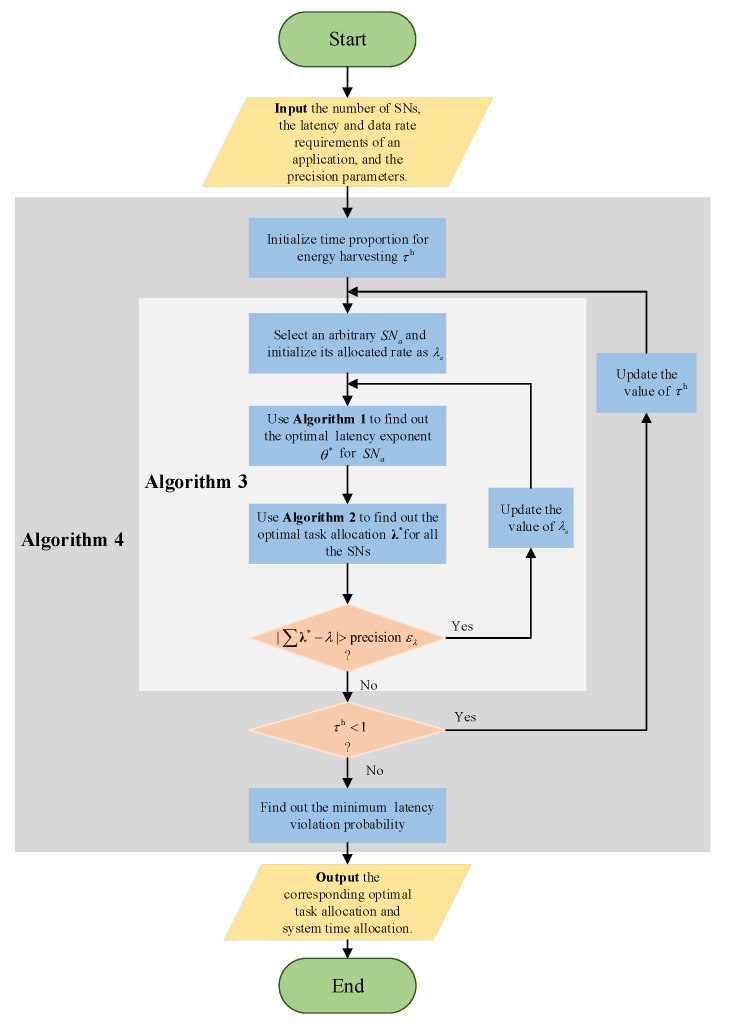
The schematic diagram of the overall scheme.

In summary, a schematic diagram is presented to introduce our proposed scheme and the relationships between different algorithms, as depicted in Figure 3.

## 6. Numerical Results

In this section, numerical results are presented and discussed. If not otherwise highlighted, the various involved parameters and the adopted analysis scenarios are as follows. The transmission power of the PS is set to $p_{0}=40$ dBm (i.e., 10 W). The length of each time block is set to T = 10 ms. The total bandwidth of the network is set to $B_{\mathrm{tot}}=20$ MHz. The power spectral density of the background noise $N_{0}=-130$ dBm/Hz. The data rate and the latency requirements of the application are set to λ=2 Mbps and $D_{\max}=100$ ms, respectively. The number of the SNs is set to K=5. For any 1≤k≤K, the energy harvesting parameters are set as $\pi_{k}=0.01$ mW, $\nu_{k}=47.083\times 10^{3}$ and $\gamma_{k}=0.0029$ mW [31]. In addition, the channel gain due to small-scaling fading between each node and PS and that between each node and BS are both assumed to follow Rayleigh distribution with mean 1. The distance between each node and the PS and that between each node and the BS are all set to $\rho_{k}=10$ m. Additionally, the path loss is assumed to be $l_{k}^{h}=l_{k}^{t}=\rho_{k}^{-2}$ with 30 dB power attenuation at a reference distance of 1 m. More intuitively, the fixed parameters are listed in Table 1.

**Figure 4:**
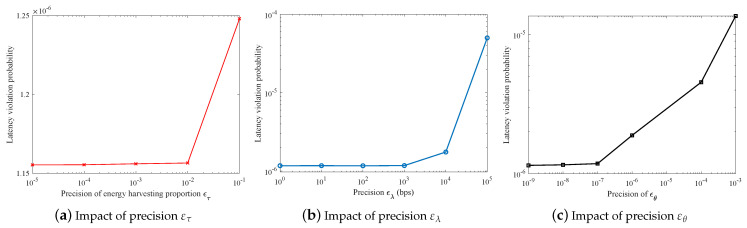
Impact of precision parameters on the numerical results.

According to Algorithms 1–4, the precision of analytical results as well as the computation complexity of the proposed resource allocation scheme both depend on the precision parameters $\varepsilon_{\tau }$, $\varepsilon_{\lambda }$, and $\varepsilon_{\theta }$. Specifically, the lower values $\varepsilon_{\tau }$, $\varepsilon_{\lambda }$, and $\varepsilon_{\theta }$ hold, the higher precision can be guaranteed for the analytical results. However, the computation complexity of the proposed scheme will increase. Hence, we first determine appropriate parameters for the subsequent numerical analysis. Figure 4 depicts the impacts of precision parameters on the network LVP. Note that, when we aim to find out the appropriate value for one type of the precision parameter, we set the other two types of precision parameters to a sufficiently low value (e.g., $\varepsilon_{\lambda }=1$ bps). It is observed that the analytical results can be convergent for each type of precision parameter. According to Figure 4, we set the precision parameters as $\varepsilon_{\tau }=0.01$, $\varepsilon_{\lambda }=1$ bps and $\varepsilon_{\theta }=10^{-7}$, respectively. Based on such configuration, a good trade-off between the analytical precision and the computation complexity can be achieved.

**Figure 5:**
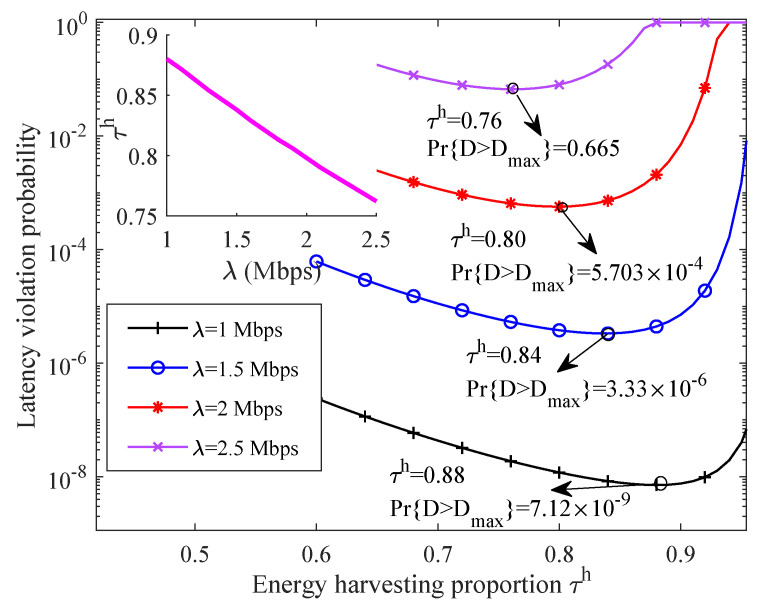
The relationship between network LVP and energy harvesting proportion under different data rate requirements.

Figure 5 depicts the relationship between network LVP and energy harvesting proportion under different data rate requirements. It is found that the network LVP first decreases with $\tau^{h}$ and then increases after reaching a certain valve, which implies that there is an optimal energy harvesting time solution for any case. The reason is that, when $\tau^{h}$ is small, the cooperative SNs need more energy to support their transmissions. Hence, the network LVP is improved as $\tau^{h}$ increases. However, when $\tau^{h}$ is large enough, increasing $\tau^{h}$ leads to shorter time to transmit data, which degrades the network LVP. In addition, the network LVP increases with application data rate requirements, since a higher source data rate is needed for each SN. In particular, when λ is small enough, it is verified that a wireless link can also guarantee an ultra-high reliable transmission for time-sensitive application—while, for the optimal energy harvesting time proportion and the application data rate requirement, we find that there is a linear relationship between them. This phenomenon is verified by the subfigure of Figure 5. The observation can help us to quickly choose the optimal energy harvesting time for other applications, which further reduces the complexity of the proposed scheme.

**Figure 6:**
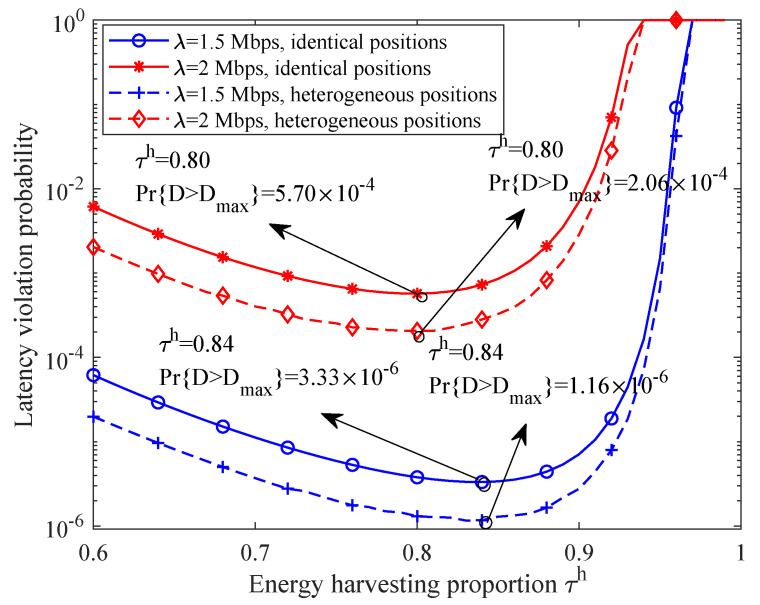
The relationship between network LVP and energy harvesting proportion under different positions of SNs.

Figure 6 depicts relationship between network LVP and energy harvesting proportion under different positions of SNs. For the SNs with heterogeneous positions, the distance between them and the PS and that between them and the BS are both set to {8,9,10,11,12} m, which guarantees the average distance as 10 m. It is observed that optimal system time configuration also exists when the positions of the SNs are different. Interestingly, when the application data rate is fixed, the optimal energy harvesting time proportion under the scenario with heterogeneous node positions is equal to that under the scenario with identical node positions. Another insightful phenomenon is observed in which the network LVP with heterogeneous node positions outperforms that with identical node positions when other conditions are fixed. This implies that a node closer to the PS and BS can sustain a higher source data rate and guarantees higher performance gain compared with the performance degradation brought by the further SN.

**Figure 7:**
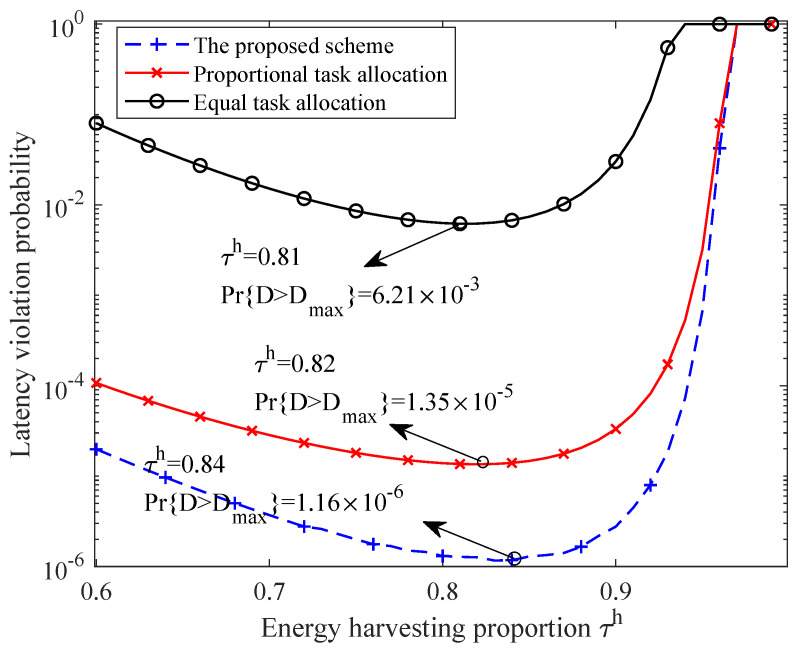
Performance comparison between the proposed scheme and the baseline schemes.

In Figure 7, we compare the LVP performance of the proposed scheme with two baseline schemes. In the scheme of proportional task allocation, the sensing data rate of a task is determined according to the channel capacity of a sensor node; it holds there as

(21) $$\lambda_{k}=\frac{E[R_{k}\left(i\right)]}{\sum_{a=1}^{K}E\left[R_{a}\left(i\right)\right]}\lambda .$$

The intuition of such scheme is that the higher data rate is allocated to the node with a better channel state. In the scheme of equal task allocation, the sensing data rate is allocated to each node equally. In addition, the system configuration is the same as Figure 6. It is observed that the proposed scheme guarantees the lowest LVP while the performance of the scheme of equal task allocation is much worse than that of the other two schemes. Moreover, the optimal energy harvesting time is different under those three schemes. Therefore, the effectiveness of the proposed scheme is validated.

**Figure 8:**
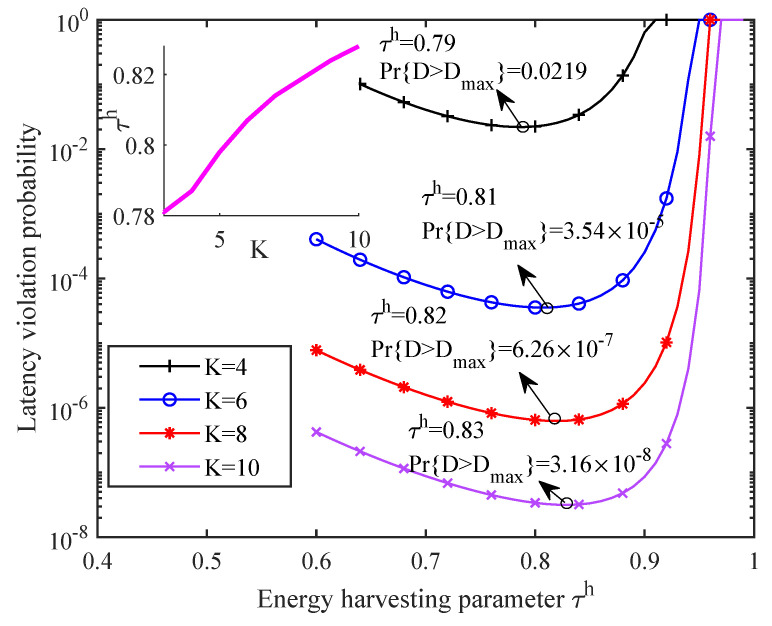
The relationship between network LVP and energy harvesting proportion under a different number of SNs.

The impact of the number of SNs on the network LVP and the energy harvesting proportion is depicted in Figure 8. When other conditions are identical, more cooperative SNs can guarantee lower network LVP. The reason is that each node needs to support a lower source rate when the number of SNs increases. In addition, we also observe that the optimal energy harvesting time proportion $\tau^{h}$ increases with the number of SNs. This is because the source data rate requirement of each node decreases with the number of SNs. As a result, less time is needed by each SN to transmit data, which naturally leaves more time to harvest energy. Moreover, we are also interested in the relationship between the optimal $\tau^{h}$ and the number of SNs. The subfigure shows that they follow an approximately linear relationship. Such observation can bring a useful guideline to determine how much time should be allocated to harvest energy when the number of nodes varies.

**Figure 9:**
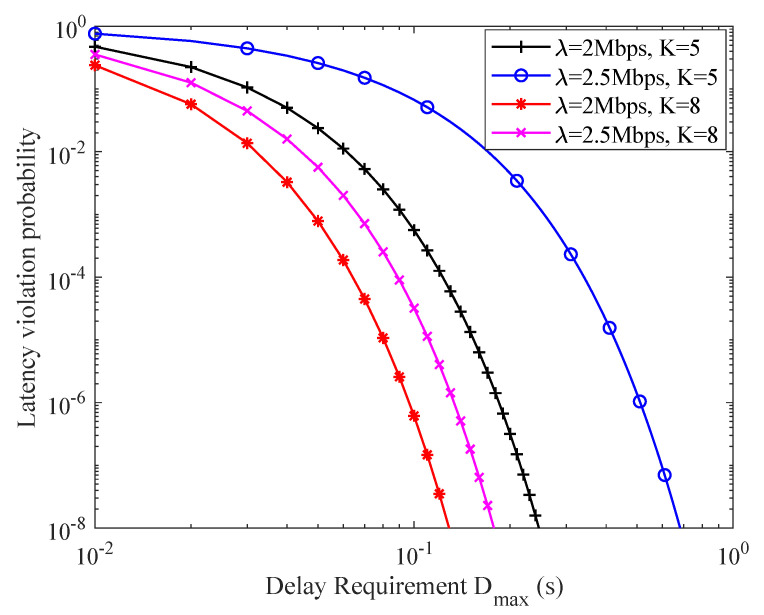
The relationship between network LVP and latency requirements.

Figure 9 illustrates the relationship between network LVP and application latency requirement. It is found that the network LVP decreases as $D_{\max}$ increases under when the application data rate requirement and the number of SNs are fixed. This is because that larger $D_{\max}$ means a looser performance requirement needed to be guaranteed by the network. Hence, the network LVP can be improved as shown in Equation (10).

**Figure 10:**
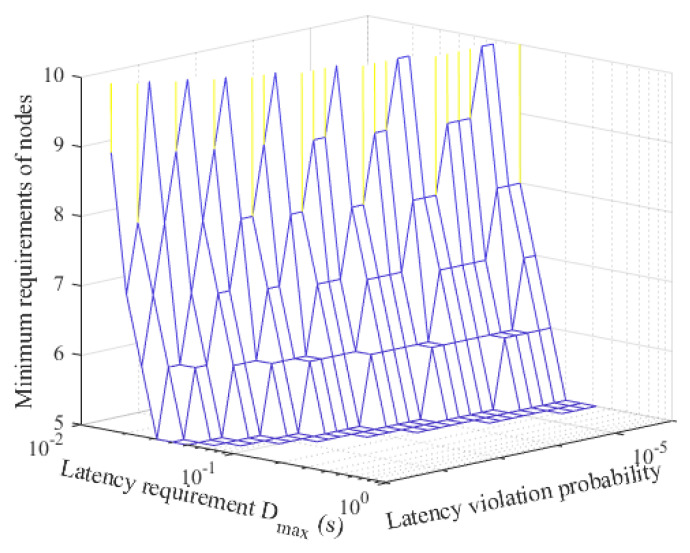
The minimum number requirements of nodes under different application latency requirements.

Figure 10 depicts the minimum number requirements of nodes under different application latency requirements. It is observed that the minimum number of requirements of SNs increases as the application latency requirement becomes tighter. With the analysis in this paper, the network operator can flexibly determine the number of SNs to serve an application in terms of data rate and latency requirements.

Additionally, we are interested in the relationship between the network LVP and energy efficiency since energy efficiency is also an important performance metric in WSNs. More specifically, as the SNs can only be powered by the power station, the network energy efficiency can be defined as

(22) $$ee=\frac{\lambda T}{p_{0}\tau^{h}TB}=\frac{\lambda }{p_{0}\tau^{h}B_{\mathrm{tot}}}.$$

**Figure 11:**
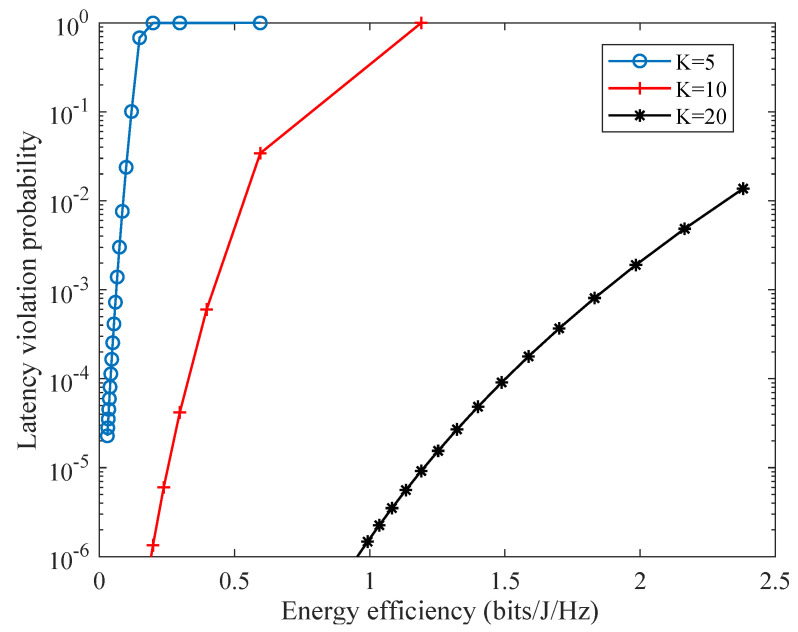
Relationship between latency violation probability and energy efficiency.

As depicted in Figure 11, the network LVP is positively related to the network energy efficiency when λ is fixed. The reason is that higher energy efficiency requires lower transmission power of the power station, which degrades the network latency performance. Hence, it is necessary to balance the requirements of network LVP and energy efficiency. In addition to the network LVP (as shown in Figure 9), the network energy efficiency can be improved through increasing the number of SNs when the total network resources are fixed. Hence, multiplexing gain is validated under the proposed scheme.

## 7. Discussion

From the numerical results and analysis, the relationship between the LVP and the energy harvesting time configuration is revealed. In addition, the impacts of application rate requirement, the delay requirement, and the number of the SNs on such relationship are depicted. To be specific, the optimal energy harvesting time linearly or nearly linearly varies with the application rate requirement and the number of the SNs. The higher application requirement or the smaller number of SNs is, the less time is allocated to the SNs to harvesting RF energy. The reason is that the SNs need more time to transmit data if the traffic load on them are heavier. According to the linear phenomenon observed in this paper, optimal energy harvesting time can be determined quickly. Therefore, the analysis can be applied to the practical wireless powered virtualized sensor networks to perform resource allocations.

Additionally, the proposed scheme can guarantee low LVP without strict resource requirements, which confirms its ability for a reliability guarantee. Particularly, while comparing with the proportional task allocation scheme and the equal task allocation scheme, the proposed scheme lowers the latency violation probability to 11.6 times and 4600 times, respectively. This is because the proposed scheme takes the heterogeneous transmission ability of each SN into account. As a result, the task rate allocated to each SN can achieve our aim that the minimum individual latency violation provability is minimized. Moreover, as discussed before, the computation complexity of the proposed scheme is $O(\frac{K}{\varepsilon_{\tau }}\log_{2}\left(\frac{\lambda }{\varepsilon_{\lambda }}\right)\log_{2}\left(\frac{1}{\varepsilon_{\theta }}\right))$. Therefore, the complexity increases linearly with the number of SNs and increases logarithmically with the accuracy requirement, which is controllable in practical networks.

## 8. Conclusions

In this paper, a stochastic latency guarantee strategy was studied in the wireless powered virtualized sensor network. A cooperative sensing framework was constructed, and a joint task and resource optimization problem was formulated. In addition, the network latency violation probability under the FDMA mode and that under the TDMA mode were proved to be identical. In addition, a bisection searching approach was proposed to find out the optimal task allocation and a one-dimensional searching scheme was proposed to find out the optimal energy harvesting time. Moreover, the proposed scheme was evaluated under different scenarios. The analysis in this paper sheds new insights on task and resource management, which can help the network operator to guarantee the application requirements in terms of data rate and latency flexibly.

## Figures and Tables

**Table 1:** Parameter settings.

Parameters	Value
the transmission power of the PS ($p_{0}$)	40 dBm (10 W)
the length of each time block (*T*)	10 ms
the total bandwidth ($B_{\mathrm{tot}}$)	20 MHz
the power spectral density of the noise ($N_{0}$)	−130 dBm/Hz
the application data rate requirement (λ)	2 Mbps
the application latency requirement ($D_{\max}$)	100 ms
the number of the SNs (*K*)	2–10
the maximum energy conversion rate ($\pi_{k}$)	0.01 mW
the circuit sensitivity ($\nu_{k}$)	$47.083\times 10^{3}$
the current leakage ($\gamma_{k}$)	0.0029 mW
the mean of Rayleigh distribution	1
the path loss between PS and $SN_{k}$ in 1m ($l_{k}^{h}$)	30 dB
the path loss between $SN_{k}$ and BS in 1m ($l_{k}^{t}$)	30 dB
precision $\varepsilon_{\tau }$	0.01
precision $\varepsilon_{\lambda }$	$10^{3}$ bps
precision $\varepsilon_{\theta }$	$10^{-7}$

## References

[1] Hamdan S., Ayyash M., Almajali S. (2020). Edge-Computing Architectures for Internet of Things Applications: A Survey. Sensors.

[2] Wang R., Liu H., Wang H., Yang Q., Wu D. (2019). Distributed Security Architecture Based on Blockchain for Connected Health: Architecture, Challenges and Approaches. IEEE Wirel. Commun..

[3] Ghori M.R., Wan T.-C., Sodhy G.C. (2020). Bluetooth Low Energy Mesh Networks: Survey of Communication and Security Protocols. Sensors.

[4] Wu D., Han X., Yang Z., Wang R. (2020). Exploiting Transfer Learning for Emotion Recognition under Cloud-Edge-Client Collaborations. IEEE J. Sel. Areas Commun..

[5] Khan I., Belqasmi F., Glitho R., Crespi N., Morrow M., Polakos P. (2016). Wireless Sensor Network Virtualization: A survey. IEEE Commun. Surv. Tutor..

[6] Su J., Chen Y., Sheng Z., Huang Z., Liu A. (2020). From M-Ary Query to Bit Query: A New Strategy for Efficient Large-Scale RFID Identification. IEEE Trans. Commun..

[7] Krikidis I. (2019). Average Age of Information in Wireless Powered Sensor Networks. IEEE Wirel. Commun. Lett..

[8] Zhang H., Guo Y., Zhong Z., Wu W. (2019). Cooperative Integration of RF Energy Harvesting and Dedicated WPT for Wireless Sensor Networks. IEEE Microw. Wirel. Components Lett..

[9] Guo M., Li L., Guan Q. (2019). Energy-Efficient and Delay-Guaranteed Workload Allocation in IoT-Edge-Cloud Computing Systems. IEEE Access.

[10] Alaslani M., Nawab F., Shihada B. (2019). Blockchain in IoT Systems: End-to-End Delay Evaluation. IEEE Internet Things J..

[11] Nkomo M., Hancke G.P., Abu-Mahfouz A.M., Sinha S., Onumanyi A.J. (2018). Overlay Virtualized Wireless Sensor Networks for Application in Industrial Internet of Things: A Review. Sensors.

[12] Haque I., Nurujjaman M., Harms J., Abu-Ghazaleh N. (2019). SDSense: An Agile and Flexible SDN-Based Framework for Wireless Sensor Networks. IEEE Trans. Veh. Technol..

[13] Gul O., Demirekler M. (2018). Asymptotically Throughput Optimal Scheduling for Energy Harvesting Wireless Sensor Networks. IEEE Access.

[14] Misra S., Chakraborty S. (2019). QoS-Aware Dispersed Dynamic Mapping of Virtual Sensors in Sensor-Cloud. IEEE Trans. Serv. Comput..

[15] Raee M., Naboulsi D., Glitho R. Energy Efficient Task Assignment in Virtualized Wireless Sensor Networks. Proceedings of the 2018 IEEE Symposium on Computers and Communications (ISCC).

[16] Zhou Y., Yu F.R., Chen J., Kuo Y. (2019). Robust Energy-Efficient Resource Allocation for IoT-Powered Cyber-Physical-Social Smart Systems With Virtualization. IEEE Internet Things J..

[17] Katona R., Cionca V., O’Shea D., Pesch D. (2020). Virtual Network Embedding for Wireless Sensor Networks Time Efficient QoS/QoI Aware Approach. IEEE Internet Things J..

[18] Choi K.W., Ginting L., Aziz A.A., Setiawan D., Park J.H., Hwang S.I., Kang D.S., Chung M.Y., Kim D.I. (2019). Toward Realization of Long-Range Wireless-Powered Sensor Networks. IEEE Wirel. Commun..

[19] Guo S., Shi Y., Yang Y., Xiao B. (2018). Energy Efficiency Maximization in Mobile Wireless Energy Harvesting Sensor Networks. IEEE Trans. Mob. Comput..

[20] Azarhava H., Musevi Niya J. (2020). Energy Efficient Resource Allocation in Wireless Energy Harvesting Sensor Networks. IEEE Wirel. Commun. Lett..

[21] Gupta V., De S. (2020). Collaborative Multi-Sensing in Energy Harvesting Wireless Sensor Networks. IEEE Trans. Signal Inf. Process. Netw..

[22] Chu Z., Zhou F., Zhu Z., Hu R.Q., Xiao P. (2018). Wireless Powered Sensor Networks for Internet of Things: Maximum Throughput and Optimal Power Allocation. IEEE Internet Things J..

[23] Anwar A.H., Seddik K.G., ElBatt T., Zahran A.H. (2016). Effective Capacity of Delay-Constrained Cognitive Radio Links Exploiting Primary Feedback. IEEE Trans. Veh. Technol..

[24] Guo C., Liang L., Li G. (2019). Resource Allocation for Low-Latency Vehicular Communications: An Effective Capacity Perspective. IEEE J. Sel. Areas Commun..

[25] Gao Y., Cheng W., Zhang H., Li Z. (2017). Heterogeneous Statistical QoS Provisioning Over Wireless Powered Sensor Networks. IEEE Access.

[26] Fidler M., Rizk A. (2015). A Guide to the Stochastic Network Calculus. IEEE Commun. Surv. Tutor..

[27] Zoppi S., Van Bemten A., Gursu H.M., Vilgelm M., Guck J., Kellerer W. (2018). Achieving Hybrid Wired/Wireless Industrial Networks With WDetServ: Reliability-Based Scheduling for Delay Guarantees. IEEE Trans. Ind. Inform..

[28] Boshkovska E., Ng D., Zlatanov N., Koelpin A., Schober R. (2017). Robust Resource Allocation for MIMO Wireless Powered Communication Networks Based on a Non-Linear EH Model. IEEE Trans. Commun..

[29] Morsi R., Boshkovska E., Ramadan E., Ng D., Schober R. On the Performance of Wireless Powered Communication with Non-Linear Energy Harvesting. Proceedings of the 2017 IEEE 18th International Workshop on Signal Processing Advances in Wireless Communications.

[30] Wu D., Negi R. (2003). Effective Capacity: A Wireless Link Model for Support of Quality of Service. IEEE Trans. Wirel. Commun..

[31] Li Z., Jiang Y., Gao Y., Sang L., Yang D. (2019). On Buffer-Constrained Throughput of a Wireless-Powered Communication System. IEEE J. Sel. Areas Commun..

